# Tablets of “Hydrochlorothiazide in Cyclodextrin in Nanoclay”: A New Nanohybrid System with Enhanced Dissolution Properties

**DOI:** 10.3390/pharmaceutics12020104

**Published:** 2020-01-28

**Authors:** Francesca Maestrelli, Marzia Cirri, Fátima García-Villén, Ana Borrego-Sánchez, César Viseras Iborra, Paola Mura

**Affiliations:** 1 Department of Chemistry, University of Florence, via Schiff 6, Sesto Fiorentino, 50019 Florence, Italy; francesca.maestrelli@unifi.it (F.M.); paola.mura@unifi.it (P.M.); 2 Department of Pharmacy and Pharmaceutical Technology, University of Granada, Campus de Cartuja, s/n 18071 Granada, Spain; fgarvillen@ugr.es (F.G.-V.); cviseras@ugr.es (C.V.I.); 3 Andalusian Institute of Earth Sciences, CSIC-University of Granada, Avda. de Las Palmeras 4, 18100 Armilla (Granada), Spain; anaborrego@iact.ugr-csic.es

**Keywords:** hydrochlorothiazide, cyclodextrins, sepiolite, nanoclay, dissolution rate, tablet

## Abstract

Hydrochlorothiazide (HCT), a Biopharmaceutical Classification System (BCS) class IV drug, is characterized by low solubility and permeability, that negatively affect its oral bioavailability, reducing its therapeutic efficacy. The combined use of cyclodextrins (CDs) and nanoclays (NCs) recently proved to be a successful strategy in developing delivery systems able to merge the potential benefits of both carriers. In this work, several binary systems of CDs or NCs with the drug were obtained, using different drug:carrier ratios and preparation techniques, and characterized in solution and in solid state, to properly select the most effective system and preparation method. Then, the best CD (RAMEB) and NC (sepiolite), at the best drug:carrier ratio, was selected for preparation of the ternary system by co-evaporation and emerged as the most effective preparation method. The combined presence of RAMEB and sepiolite gave rise to a synergistic improvement of drug dissolution properties, with a two-fold increase in the amount of drug dissolved as compared with the corresponding HCT-RAMEB system, resulting in an approximately 12-fold increase in drug solubility as compared with the drug alone. The ternary system that was co-evaporated was then selected for a tablet formulation. The obtained tablets were fully characterized for technological properties and clearly revealed a better drug dissolution performance than the commercial reference tablet (Esidrex^®^).

## 1. Introduction

Hydrochlorothiazide (HCT) is a thiazide diuretic drug widely used in the treatment of heart failure, hypertension, hypovolemic shock, or edema [1]. HCT is a class IV drug, according to the Biopharmaceutical Classification System (BCS), characterized by low aqueous solubility and low permeability [2]. Due to these factors, this drug is poorly absorbed in the gastrointestinal tract, thus, resulting in a variable and low bioavailability after oral administration [3]. Moreover, HCT can be easily hydrolyzed in an aqueous environment, which also poses stability problems [4].

Cyclodextrins (CDs) are a family of cyclic oligosaccharides with a hydrophilic outer surface and a lipophilic central cavity used as complexing agents to increase aqueous solubility of poorly soluble drugs and to increase their bioavailability and stability. The natural CDs, named α−, β−, and γCD consist of six, seven, or eight glucopyranose units and among them, βCD usually demonstrates the higher complexing ability toward drugs [5]. Cyclodextrins (CDs) have been successfully used as carriers to improve the solubility and bioavailability of antihypertensive agents such as HCT [6]. CD complexation can provide additional advantages, including taste masking, increased drug stability, and drug permeation, thus, leading to dose lowering and reduction of side effects [7]. Mendes et al., 2016 showed the capacity of βCD to increase HCT solubility, protect the drug from hydrolysis and enhance the in vivo effect [8]. Moreover, the effectiveness of βCD in increasing drug permeability across the small intestine of rats was recently demonstrated by Altamimi et al., 2018 [9]. However, βCD has relatively low solubility and often cannot be used at concentrations suitable for pharmaceutical applications. For these reasons several modified βCDs have been prepared, and now its hydroxypropyl, methyl, and sulfobutylether derivatives have been commercially used as new pharmaceutical excipients.

Clay minerals can be used as excipients in several medicinal products and can be effectively used in the development of new formulations. Nanoclays (NCs) are fibrous inorganic matrices that are able to entrap little molecules and release them in particular conditions and have aroused particular interest as drug carriers due to their biocompatibility, high drug loading power, low cost, and very poor toxicity [10,11]. Moreover, their ability to enhance the dissolution properties and, then, the bioavailability of poorly soluble drugs has been reported [12,13].

Tablets are still the most used solid dosage form in the market due to their manufacturing efficiency, good stability, and good patient acceptance [14]; and CD and fibrous clays used as excipients for tablet formulation is well recognized and consolidated [13,15]. Recently, “drug-in-CD-in-NC” hybrid systems have been developed and used to prepare tablets of oxaprozin, a poorly water-soluble NSAID, with improved dissolution properties and enhanced therapeutic effect on rats [16,17]. Taking these considerations into account, the aim of this work was to assess the effectiveness of such a particular approach, based on the combined use of CDs and NCs as drug carriers, for the development of a powerful tablet formulation for oral delivery of HCT. With this purpose, the solubilizing effect of different CDs and NCs towards the drug was first investigated, in order to select the best carriers. In fact, it is not possible to determine in advance which kind of CD or NC better improves the drug dissolution properties, as well as it is not possible to predict the effect of drug-CD complexation on the nanoclay interaction and entrapment. Moreover, in order to evaluate the influence of the preparation technique in establishing effective drug-carrier interactions, binary and ternary combinations of the drug with CDs and NCs were prepared by different methods and characterized for solid-state (by differential scanning calorimetry and X-ray powder diffractometry) and dissolution properties. The best CD and NC carriers and the most effective preparation method were then selected to obtain the hybrid ternary system, which was used for the development of tablets. These were suitably evaluated for technological properties and tested for dissolution behavior and compared with a marketed tablet formulation.

## 2. Materials and Methods

### 2.1. Materials

Hydrochlorothiazide (HCT) was a gift from Menarini (L’Aquila, Italy). β-Cyclodextrin (βCD) and hydroxypropyl-β-cyclodextrin (Kleptose HP, HPβCd) were kindly donated by Roquette (Lestrem, France). Amorphous randomly methylated-β-cyclodextrin (RAMEB, average MS 1.8) and hydroxyethyl-β-cyclodextrin (HEβCD, average MS 1.0) were a gift from Wacker-Chemie GmbH (Munchen, Germany). Sulfobutylether-β-cyclodextrin (SBEβCD) Dexolve^®^ was gifted from CycloLab (Budapest, Ungary). Sepiolite was from Vicalvaro (Spain) (SV); attapulgite (Pharmasorb colloidal, PHC) was from BASF, and bentonite (or smectite) (VeegumHS, VHS) was kindly gifted by Vanderbilt Minerals (USA). Magnesium stearate and polyvinylpyrrolidone (PVP K 30) were obtained from Sigma-Aldrich Chemie GmbH (Steinhelm, Germany). Sodium starch glycolate (Explotab^®^) was from JRS Pharma (Rosenberg, Germany). Tablets of HCT commercially available in Italy (Esidrex^®^) containing 25 mg of drug were from Novartis Farma Spa (Varese, Italy). Other chemicals and solvents were of reagent grade and used without further purification. 

### 2.2. Phase Solubility Studies

Phase-solubility studies of HCT with different cyclodextrins were performed by adding an excess of drug to phosphate buffer solutions (pH 5.5) containing increasing concentrations of CD, i.e., 0 to 12.5 mM for βCD and 0 to 25 mM for the other CD derivatives. The vials were sealed, and the suspensions were electromagnetically stirred (500 rpm) at a constant temperature (25 °C) until equilibrium (3 days). Then, an aliquot of solution was withdrawn with a filter-syringe (pore size 0.45 μm), and the drug concentration was spectrometrically determined (UV/Vis 1601 Shimadzu, Tokyo, Japan) at λ_max_ 272.2 nm. The linearity was determined on five concentration levels (from 3 to 20 mg/L). The calibration curve was *y* = 0.0623*x* + 0.003, *r*^2^ = 0.999. LOQ = 1 mg/L and LOD = 0.33 mg/L. The presence of CD did not interfere with the drug spectrophotometric assay. Each experiment was performed in triplicate (C.V. < 2.5%). The apparent stability constants of the drug–CD complexes were calculated from the slope of the linear portion of the phase-solubility diagrams and the drug solubility (S_o_) in the dissolution medium according to Equation (1) [18]:

Ks = slope/S_o_*(1 − slope) (1)

The complexation efficiency (CE) was calculated from the slope of the phase-solubility diagrams according to Equation (2) [19]:

CE = slope/(1 − slope)(2)

The solubilizing efficiency (SE) of CDs towards the drug was calculated as the ratio of drug solubility in the presence of the maximum concentration of CD used with respect to that of the drug alone.

### 2.3. Preparation of Drug–CD and Drug–NC Binary Systems

Physical mixtures (PM) were prepared by mixing equimolar amounts of drug and CD with the same granulometric size (75 to 150 μm). By submitting the PM to grinding, using a high-energy vibrational micro-mill for 30 min at 24 Hz (Mixer Mill MM 200, Retsch GmbH, Dusseldorf, Germany), co-ground binary systems were obtained. Kneaded products (KN) were prepared starting from the corresponding PM. Briefly, the PM were placed in a mortar and 0.2 mL of a water/ethanol 50:50 *v*/*v* solution were added until a dough was obtained. The mixture was then manually ground with a pestle until a powder was obtained. A complete drying was achieved putting the powder in an oven at 40 °C for 24 h.

HCT binary systems with NCs (PHC, VHS, and SV) were prepared at different w/w ratios (1:1; 1:2; 1:4) by physical mixing (i.e., PM), as described above for HCT-CD systems. Binary systems with the selected NC (SV) at the selected *w*/*w* ratio (1:4 *w*/*w*) were prepared by different techniques. Co-evaporated products (COE) were prepared by dissolving HCT in the minimum amount of ethanol and suspending SV in water, mixing and removing the solvent by rotary evaporation; the solid product was collected and left to dry in an oven for 24 h at 40 °C. Cofused products (COF) were realized by heating the PM under magnetic stirring at 300 °C for 10 min. Co-ground systems (GR) were obtained by ball-milling the PM in a high-energy vibrational micro-mill (Mixer Mill MM 200, Retsch GmbH, Dusseldorf, Germany) at 24 Hz for 30 min [10,16]. Solvent-heated products (SH) were obtained by adding 5 mL of ethanol to the PM under magnetic stirring for 5 min and heating at 300 °C for 5 min until the solvent evaporation [20,21]. Solvent-sonicated products (SS) were prepared by a partial modification of the method of Yendluri et al., 2017 [22,23]. Briefly, 20 mg of drug were dissolved in 1 mL of ethanol to obtain a saturated solution where SV was added. The mixture was sonicated with a Sonopuls HD2200 ultrasound homogenizer (Bandelin Electronic GmbH, Berlin, Germany) equipped with a KE76 probe at 50% of power for 10 min and left to dry overnight to ensure maximum loading. The day after, the sample was washed with fresh ethanol and filtered in order to remove aggregates. The sample was dried in a vacuum desiccator overnight and powdered. The solvent magnetic stirring technique (SMS) is a partial variation of the method used by Lun et al., 2014 [24]. Briefly, the saturated solution of drug in ethanol containing SV was kept under stirring overnight; then, the solid in suspension was separated by centrifugation, filtered and dried overnight at 60 °C. 

### 2.4. Preparation of Drug-CD-Sepiolite (SV) Ternary Systems

Ternary physical mixtures (PM) were prepared by 15 min tumble mixing the drug with the selected CD (HCT:RAMEB 1:1 mol/mol) and SV (HCT:SV 1:4 *w*/*w* ratio). Co-evaporated ternary products (COE) were prepared by dissolving equimolar amounts of HCT and RAMEB in the minimum amount of ethanol and water, respectively, mixing the solutions, suspending SV (HCT:SV 1:4 *w*/*w* ratio) under mixing and removing the solvent by rotary evaporation; the solid product was collected and left to dry in an oven for 24 h at 40 °C. 

### 2.5. Solid-State Characterization

Thermal analysis of pure components, drug–CD and drug-SV binary systems obtained with the different techniques was performed by a Mettler TA 4000 Star^e^ system (Mettler Toledo, Greifensee, Switzerland). About 5 to 10 mg of each sample were accurately weighed by a MX5 Microbalance (Mettler-Toledo, Greifensee, Switzerland), placed in sealed aluminum pans with pierced lid, and scanned at a heating rate of 10 °C min^−1^ under static air atmosphere, at a temperature in the range 30–300 °C. The instrument was calibrated using Indium as a standard (99.98% purity, melting point 156.61 °C, and fusion enthalpy 28.71 J/g). 

The relative degree of drug crystallinity (RDC) in the samples was calculated according to Equation (3) [17]:

(3) $$RDC=\frac{\Delta Hsample}{\Delta Hdrug}\times 100\%$$

where Δ*H sample* and Δ*H drug* are, respectively, the heat of fusion of the drug in the sample (normalized to the drug content) and of the pure drug. Measurements were performed in triplicate and the relative standard deviation of crystallinity data was about ±4% to 5%.

The X-ray powder diffraction analysis (XRPD) was conducted at room temperature (2θ = 5 to 30° and scan rate = 0.05°/s) with Bruker D8-advance apparatus (Silberstreifen, Germany) at a 40 mV voltage and 55 mA current using a Cu Kα radiation and a graphite monochromator.

### 2.6. Dissolution Rate Studies

Dissolution rate studies of HCT as such and from its different binary and ternary systems as well as from the final tablets were performed according to the dispersed amount method. 

Powder samples, containing a fixed amount of drug, were sieved and the selected granulometric fraction (75 to 150 μm) was placed into a 150 mL beaker containing 75 mL of pH 5.5 phosphate buffer solution. In the beaker, thermostated at 37 ± 0.5 °C, a three-blade paddle (1.5 cm radius) was centrally placed and rotated at 100 rpm. Drug content was determined as described above (Section 2.1) in samples (3 mL) periodically withdrawn and filtered with a syringe-filter (pore size 0.45 μm). Fresh medium was added to replace the sampling and a correction was made for the cumulative dilution. 

Each test was repeated four times (C.V. < 5%). All data were analyzed by ANOVA (one-way analysis of variance) (GraphPad Prism version 4.0 program, Inc. San Diego, CA, USA). Differences were considered statistically significant when *p* values were <0.05.

### 2.7. Tablet Formulation and Characterization 

Flat tablets containing 25 mg of drug were prepared with the selected ternary system (COE HCT-RAMEB-SV). In order to obtain mixtures with proper flowability, disintegration, and compactability properties, 4% sodium starch glycolate (Explotab^®^) as superdisintegrant, 10% of polyvinylpirrolidone (PVP K30) as binder, and 1% of Mg stearate as lubricant were combined to the drug or drug-carrier systems. After a mixing of 15 min in a turbula mixer, the powders were compressed at 2.5 tons for 3 min using a hydraulic press. Uniformity of content, weight, diameter, thickness, hardness, friability, and disintegration tests were performed according to European Pharmacopoeia (Ph. Eur.) 9th Edition. In order to obtain results comparable with the powders, dissolution studies were instead performed according to the method described above (Section 2.5).

The same test was also performed on Esidrex®, an HCT tablet formulation present on the market.

## 3. Results and Discussion

### 3.1. Phase Solubility Studies

**Figure 1:**
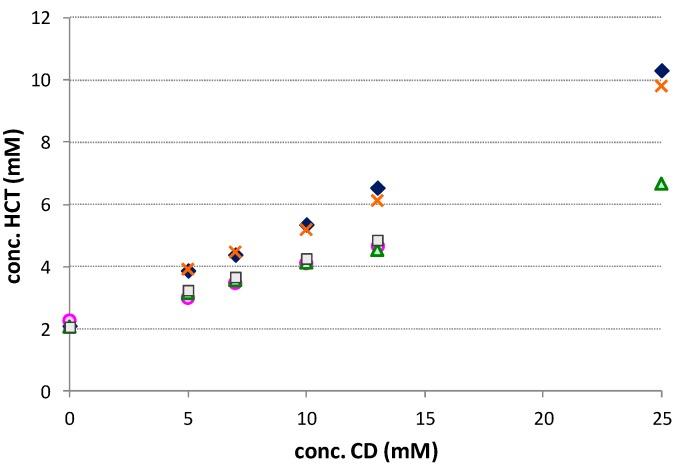
Phase solubility diagrams of HCT with βCD(□), HPβCD (Δ), HEβCD (○), SBEβCD (X), and RAMEB (◆). Each value represents the mean of 9 measurements.

The phase solubility studies evidenced a linear increase of drug solubility with increasing CDs concentration (A_L_ type), as reported in Figure 1. The results indicate, in all cases, the formation of soluble complexes of probable 1:1 mol:mol stoichiometry [18]. The parameters, reported in Table 1, evidenced the difference in the solubilizing and complexing power towards HCT among the different CDs. 

Our results were in full agreement with those of Onnainty et al., 2013 [25], who reported the same type of phase-solubility diagrams for HCT complexation with βCD. The highest values of complex apparent stability constant (K_1:1_), complexation efficiency (CE), and solubilizing efficiency (SE) were obtained with RAMEB and SBEβCD that gave rise to an approximately five-fold increase in drug solubility as compared with the drug alone (Table 1). The better performance of these derivatives can be attributed to the presence of methyl and sulfobutylether substituents which extended the CD hydrophobic region and improved substrate binding via a hydrophobic effect, as just observed for other lipophilic drugs [16,26].

### 3.2. Characterization of the Drug–CD Binary Systems

**Figure 2:**
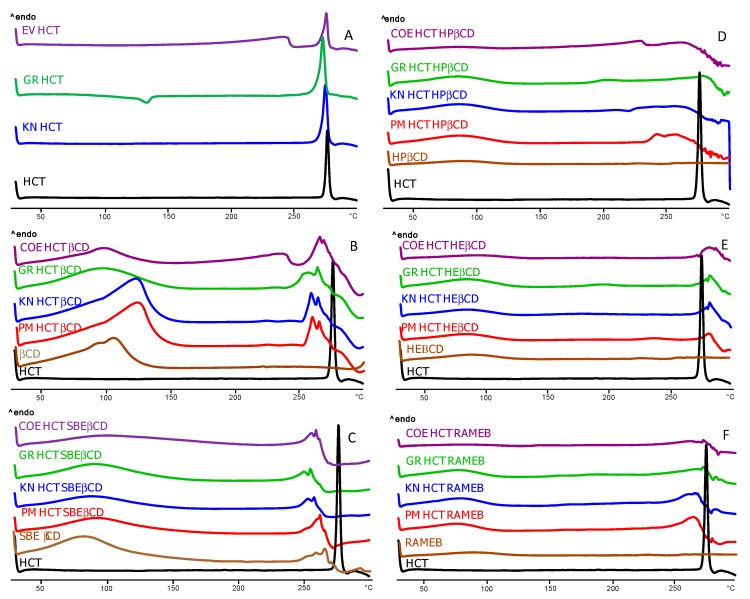
DSC curves of pure HCT (**A**) untreated or submitted to kneading (KN), grinding (GR) or dissolution-solvent evaporation (EV) and its binary systems with βCD (**B**), HPβCD (**C**), HEβCD (**D**), SBEβCD (**E**), and RAMEB (**F**), obtained by physical mixing (i.e., PM), co-grinding (GR), kneading (KN), and co-evaporation (COE).

DSC curves of pure HCT and CDs and of their binary systems obtained with the different techniques were recorded in order to investigate the effect of the CD type and preparation technique towards drug amorphization and complexation (Figure 2A). Thermograms of pure drug submitted to the different treatments used for the preparation of the binary systems were also recorded, in order to evidence any effect of the preparation technique on drug solid-state properties. 

The drug alone thermogram showed a sharp melting peak (T_peak_ = 274.4 °C and ΔH = 152.8 J·g^−1^), as expected for an anhydrous crystalline compound. The kneading does not seem to affect the thermal behavior, causing just a slight decrease in T_peak_ (272.2 °C) and RDC (98.96%).

The thermal curve of the drug submitted to the grinding process showed an exothermic phenomenon at 134.5 °C which is attributed to the recrystallization of the drug fraction amorphized during the grinding procedure; it was followed by the melting peak at 271.2 °C, whose reduced intensity (ΔH_fus_128.1 Jg^−1^) indicated some loss of crystallinity (RDC 83.23%). In the evaporated product, the drug melting peak at 274.1 °C was characterized by a sensible reduction of fusion enthalpy (72.1 Jg^−1^). The calculated RDC of 46% suggested an appreciable drug amorphization, as a consequence of its dissolution in the water-ethanol mixture and the following solvent evaporation.

In Figure 2B, the thermal profiles of HCT binary systems with βCD are reported. The DSC curve of βCD in the examined temperature range was characterized by an intense and broad endothermic effect that ranged between 50 to 130 °C, due to its dehydration. The PM showed the CD dehydration band, followed at higher temperatures by an endothermic event characterized by the presence of two peaks at 265 °C and 269 °C. These can be attributed to the partial superimposition of the drug melting and the CD thermal decomposition, both shifted at lower temperature than the corresponding pure components, due to their co-presence, as observed also by other authors [27,28]. In fact, CD alone started to decompose usually over 300 °C [29]. A thermal behavior substantially similar to that of PM was found for GR, KN, or COE products, suggesting poor host–guest interactions. A rather similar behavior (Figure 2C) was observed for the series of products with SBEβCD. As previously observed by Cirri et al., 2017, the thermal profile of this CD showed a broad endothermic effect between 60 to 110 °C due to its dehydration, and another endothermic band between 250 and 280 °C, due to degradation phenomena [30]. All binary systems prepared with the different techniques showed the superimposition of HCT melting and CD decomposition phenomena, similar to that observed in the simple PM. As for the thermal profiles of binary systems with HPβCD, HEβCD and RAMEB (Figure 2D–F, respectively) after the initial broad endothermic effect, due to the amorphous CD dehydration, the drug and CD endothermic decomposition phenomena in the range 250–260 °C was still barely detectable only for the PM with HPβCD, while the drug melting peak completely disappeared in all other systems, where only the CD decomposition band was observed.

**Figure 3:**
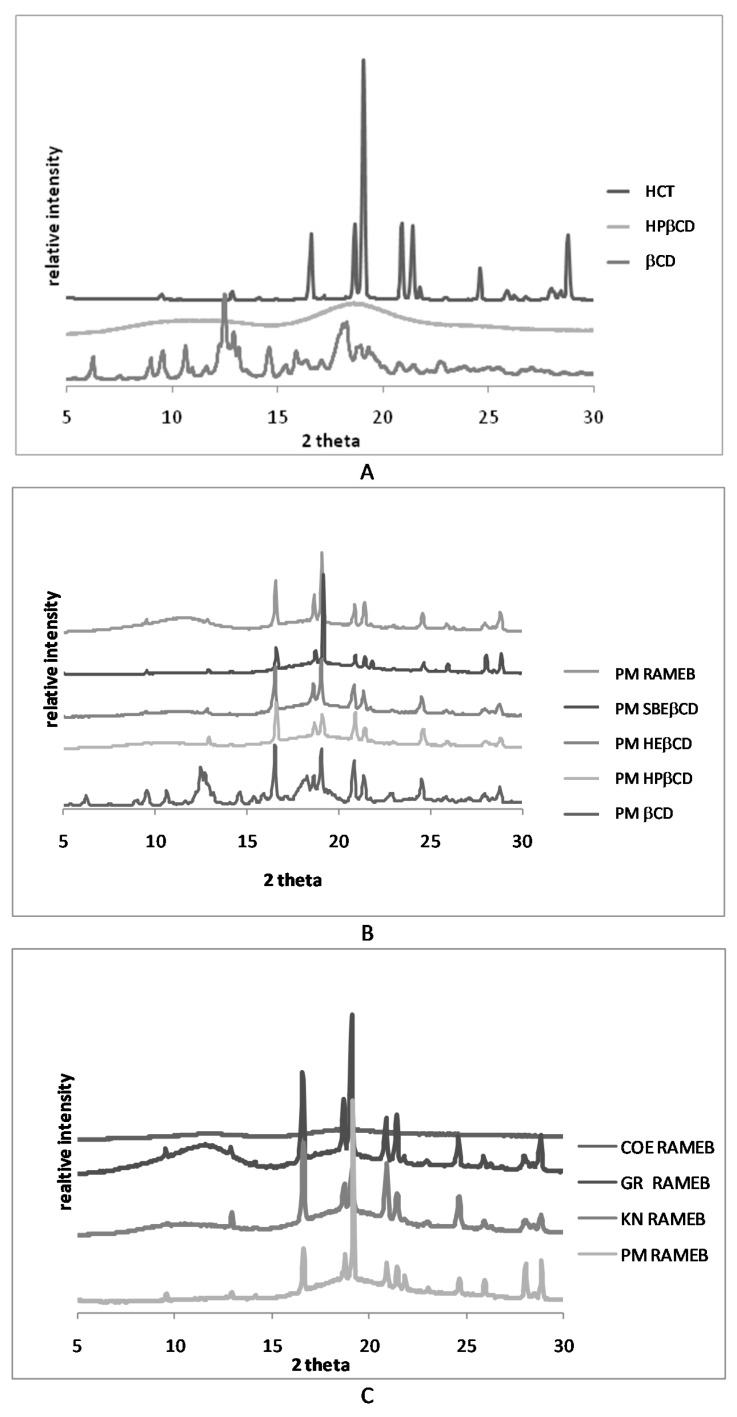
XRPD patterns of pure HCT and CDs (**A**) and of equimolar physical mixtures (PM) (**B**) with all CDs and the co-ground (GR), kneaded (KN), and co-evaporated (COE) products with RAMEB (**C**).

In order to better elucidate the DSC findings and, in particular, to evidence any possible artifact of the technique, due to a heating-induced interaction between the components as a consequence of the thermal energy supplied to the sample during the DSC scans [31], XRPD analysis was conducted. As shown in Figure 3A, the patterns of pure drug and βCD presented several sharp peaks, characteristic of crystalline substances, whereas all βCD derivatives, as shown, as example, for HPβCD, presented an almost flat pattern typical of amorphous substances. Representative drug peaks were clearly detectable in the patterns of all PMs (Figure 3B), even if reduced in intensity, particularly, in combinations with the amorphous partners, indicating that any solid-state interaction occurred during the simple mixing of drug and CDs. However analogous results to those of PMs were also obtained for all binary systems with βCD and different CD derivatives, with the only exception for COE products obtained with RAMEB and SBEβCD (Figure S1, Supplementary Material). Thus, the disappearance of the drug melting peak, observed in the other cases can be attributed to heating-induced interactions due to the thermal energy supplied to the sample during the scan. However, the completely amorphous pattern exhibited by the COE products with RAMEB and SBEβCD proved that the actual drug amorphization and complexation was achieved only by this preparation technique with these CDs (Figure 3C).

**Figure 4:**
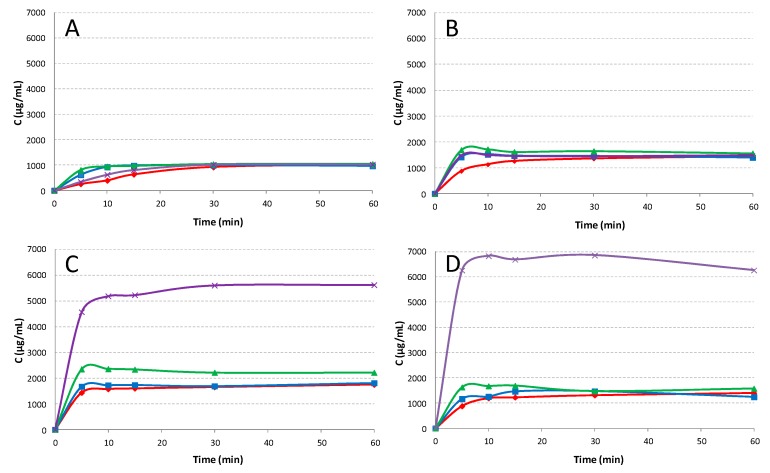
Dissolution profiles of pure HCT (**A**) untreated (◆ red line) or submitted to kneading (■ blue line), grinding (▲ green line), or dissolution-solvent evaporation (x violet line) and from its binary systems with βCD (**B**), SBEβCD(**C**), and RAMEB (**D**), obtained by physical mixing (PM) (◆ red line), kneading (KN) (■ blue line), co-grinding (GR) (▲ green line), and co-evaporation (COE) (x violet line). Each value represents the mean of 3 experiments.

The results of dissolution rate studies performed on pure drug, both untreated and submitted to the different techniques and on the different binary systems with all the CDs, are presented in Figure 4.

As shown, HCT alone (Figure 4A) always reached a solubility of 1 mg/mL, even if the treatments, especially the grinding one, initially allowed a more rapid release, probably due to a particle size reduction of the powder. In binary systems with βCD (Figure 4B) a better profile was also observed for the simple PM that reached a plateau level of 1.45 ± 0.5 mg/mL, due to the improved drug wettability and possible in situ complexation phenomena. A little more favorable effect on the drug dissolution rate, particularly in the first minutes, was observed for all treated products, in virtue of the more intimate contact between the components brought about by the sample preparation method. Similar results were obtained for drug systems with HEβCD and HPβCD (data not shown), regardless of the sample preparation technique. Instead, significant differences were observed for the series of products with SBEβCD and RAMEB (Figure 4C,D) especially for COE systems, which achieved plateau levels around 5.5 ± 0.3 and 6.8 ± 0.2 mg/mL, respectively. It should be pointed out that the COE systems with SBEβCD and RAMEB were the only ones actually containing the drug in an amorphous/complexed status, as revealed by XRPD analysis. Moreover, the results were in accordance with phase solubility studies, where SBEβCD and RAMEB showed the higher complexing and solubilizing efficiency towards HCT.

Taking into account these results, co-evaporation was selected as the most effective preparation technique and RAMEB as CD that lead to the best drug dissolution profile.

### 3.3. Characterization of Drug–NC Binary Systems 

DSC analyses were then performed on HCT binary systems with three different nanoclays (SV, PHC, and VHS) at three different drug:NC *w*/*w* ratios (1:1, 1:2, and 1:4) in order to find the nanoclay that more strongly interacts with the drug and the most suitable weight ratio between the components. The results, as summarized in Table 2, show evidence that no interaction occurred between HCT and VHS, as indicated by the absence of variations in drug melting peak or enthalpy. Evidently, on the one hand, the typical lamellar stratified structure of VHS, despite having proven to effectively entrap molecules by cation, exchange with the hydrated cations present in its interlayers [10] showed limited interaction with a lipophilic molecule as HCT. On the other hand, the typical fibrous structure of SV and PHC, consisting in hollow nanotubes, seemed to be more suitable to entrap lipophilic drugs. SV proved to have a greater interaction ability towards HCT, raising to the highest reduction in drug crystallinity at the 1:4 *w*/*w* ratio (RDC 37%) as a consequence of its better dispersion into the nanoclay structure. The higher interaction ability of SV than PHC towards HCT probably could be related to the different dimensions of their channels (0.37 × 1.06 nm for SV and 0.37 × 0.64 nm for PHC) [32]. Moreover, these findings were in accordance with those previously obtained with another lipophilic drug, oxaprozin, where SV provided the best results in terms of NC–drug interactions [16].

SV was then selected in order to test the effect of different preparation methods and experimental conditions (such as use of solvent, stirring rate, temperature, etc.) on the performance of drug-nanoclay systems. Binary systems at 1:4 *w*/*w* drug:SV ratio were prepared by co-evaporation (COE), co-fusion (COF), co-grinding (GR), solvent-heating (SH), solvent-sonication (SS), and solvent magnetic stirring (SMS) techniques. The products obtained were submitted to DSC analyses and the results are summarized in Table 3 in terms of drug melting temperature and enthalpy and percent of residual drug crystallinity (RDC %).

Even if all the used techniques gave rise to a marked reduction of intensity of the drug melting peak, with the trend PM < SS = SMS < GR < COF < SH, its complete disappearance was obtained just with the COE.

**Figure 5:**
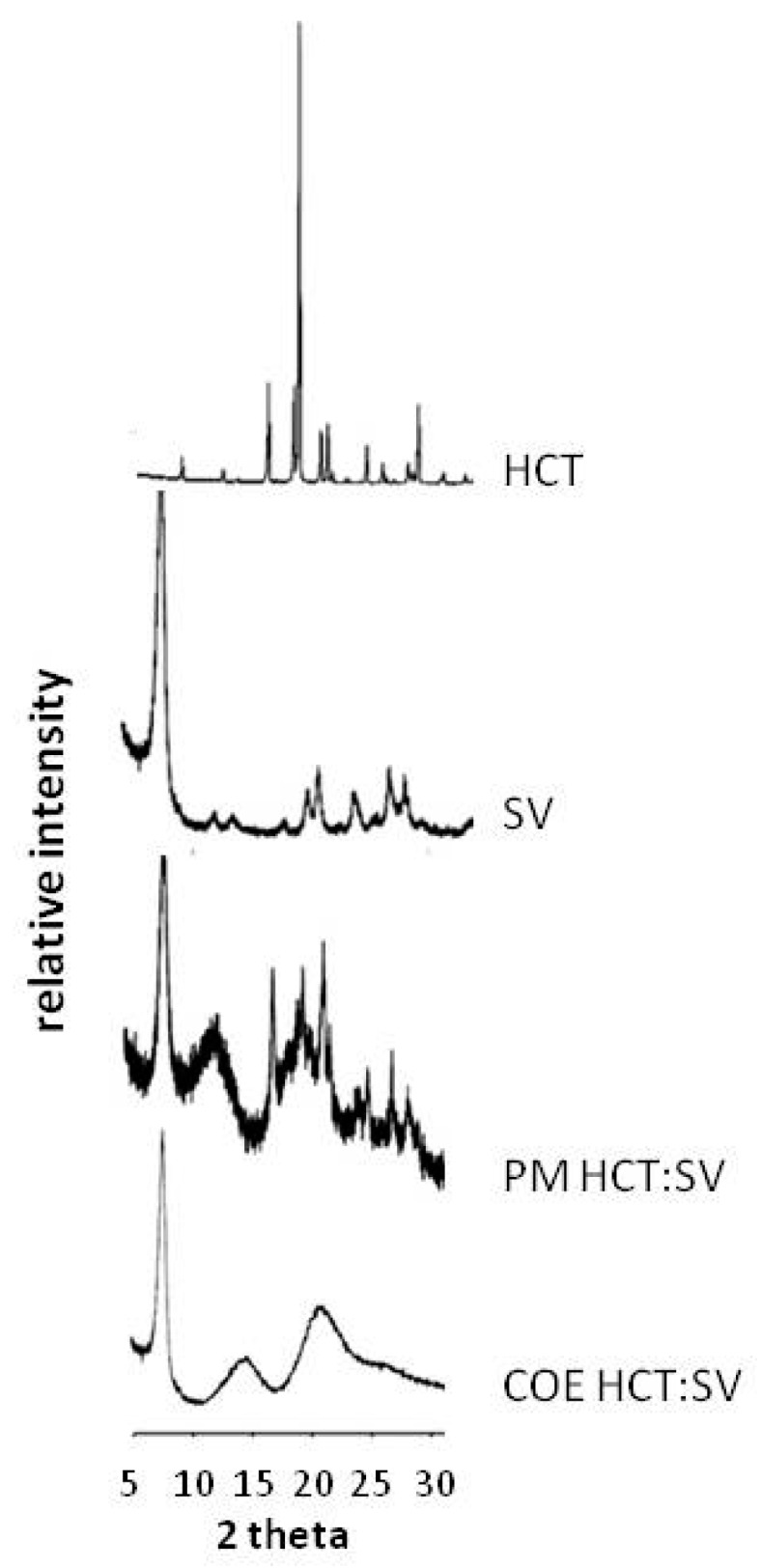
Patterns of HCT, SV, and their 1:4 *w*/*w* binary systems obtained by physical mixing (PM) and co-evaporation (COE).

The XRPD patterns of pure components, PM and COE product are reported in Figure 5. SV showed a typical crystalline pattern clearly recognizable in the PM and still detectable in the COE product with the drug. However, the crystallinity peaks of HCT were well visible in the PM and in the other binary systems (data are reported just for PM as example) while they completely disappeared in the COE product. 

On the basis of these results, ternary systems were prepared by physical mixture and co-evaporation with RAMEB (HCT-RAMEB 1:1 molar ratio) and SV (HCT-SV 1:4 *w*/*w* ratio) and compared with the corresponding binary systems. X-ray diffractograms, performed on ternary PM and COE products confirmed the complete drug amorphization in the COE product, thus, confirming the DSC results.

### 3.4. Dissolution Rate Studies

**Figure 6:**
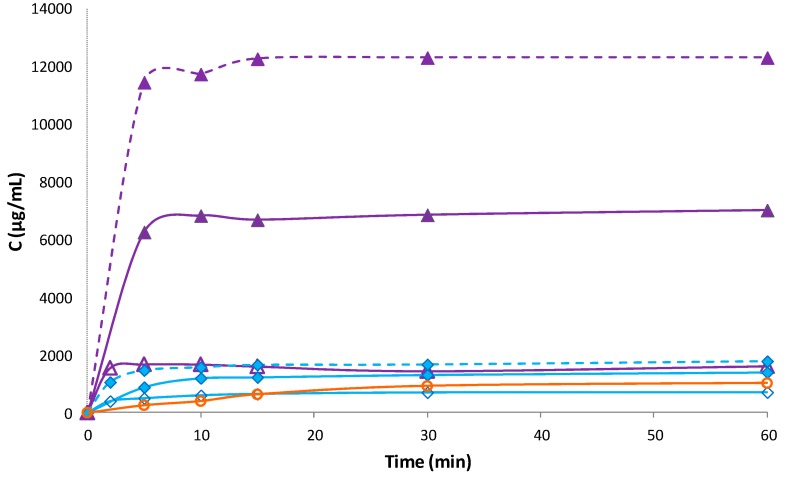
Dissolution profiles of HCT alone (o) and from its binary and ternary systems with RAMEB and SV: Physical mixtures (PM) (blue lines) with RAMEB (◆), SV (◊), and both (◆ dotted line) and co-evaporated (COE) products (violet lines) with RAMEB (▲), SV (Δ), and both (▲ dotted line). Each value represents the mean of 3 experiments.

The dissolution profiles of binary and ternary PM and COE products with RAMEB and SV are shown in Figure 6.

Binary HCT-SV systems obtained by co-evaporation revealed an increase in drug dissolution rate with respect to pure drug, more evident as compared with the simple PM, probably, due to the complete drug amorphization and its closer dispersion within the NC matrix. However, the dissolution performance of drug-SV systems was considerably worse than the corresponding binary systems with RAMEB, indicating that drug complexation with RAMEB was far more effective than NC entrapment. The simultaneous presence of both carriers in the ternary systems gave rise to a synergistic effect in improving HCT dissolution properties. This was particularly evident in the ternary COE product, which showed about a two-fold increase of the dissolved drug amount after 5 min, with respect to the binary HCT-RAMEB COE system and reached a plateau level of 12.3 ± 0.9 mg/mL. Analogous results have been previously obtained for oxaprozin [16]. However, it is necessary to evidence that, in the case of oxaprozin, the grinding technique with CDs and the cofusion technique with nanoclay resulted in the best technique to improve the drug solubility, whereas in the case of HCT, the best results were obtained with co-evaporation. These findings confirm that the combined use of cyclodextrins and nanoclays, joined with the most appropriate sample preparation technique, can be a successful strategy to strengthen the benefits related to their potential to enhane solubility and dissolution rate of lipophilic drugs. Nevertheless, it is evident that preformulation studies are important in order to find the most appropriate preparation technique.

On the basis of these findings, the HCT-SV-RAMEB COE product was selected for the development of a new tablet formulation.

### 3.5. Tablet Formulation and Characterization

Compatibility studies performed by DSC analysis demonstrated the complete compatibility between the drug and the selected tablet excipients, as shown in Figure S2 of Supplementary materials. Preliminary studies conducted on the excipients allowed the obtainment of the selected tablet composition. Particularly, it was necessary to bring the binder percentage up to 10% because tablets prepared with a lower content did not pass the friability test. Tablets prepared with COE HCT SV RAMEB were fully characterized according to Ph. Eur. 9th Edition and compared with the marketed product, Esidrex® and the tablet properties in terms of hardness, friability, and disintegration time are summarized in Table 4. The batches demonstrated a good uniformity passing the tests of content (RDS ˂ 1%), diameter (RSD < 0.3%), thickness (RSD < 1%) and weight (RSD < 3%), and uniformity. As reported, the new tablets showed higher hardness and higher disintegration time than the commercial ones, but they were within the limits of the values imposed by the Ph. Eur. 9th Edition for uncoated tablets (15 min). The SV presence was fundamental to the improvement of the powder compactability and the obtainment of tablets with acceptable hardness and low friability properties, thus, demonstrating its suitability for direct compression.

**Figure 7:**
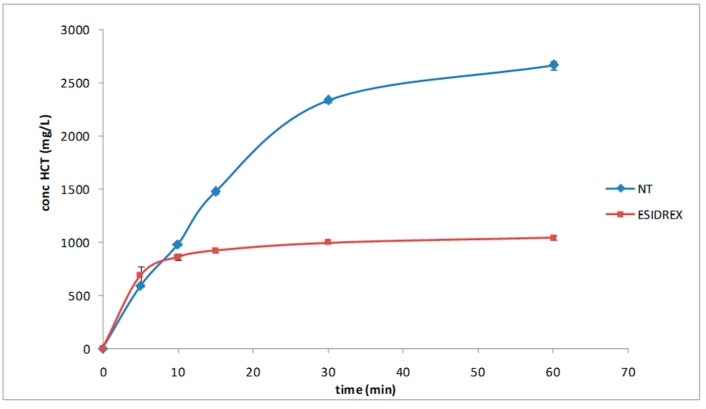
Dissolution profiles of HCT from the new tablet formulation and from the marketed tablet. The results proved the maintenance of the synergistic effect of NC and CD in improving the dissolution behavior also in the tablet formulation. Each value represents the mean of 3 measurements.

Dissolution studies were performed using the dispersed amount method with an excess drug amount in order to evidence the effectiveness of our formulation. As shown in Figure 7, the results clearly demonstrated the better dissolution profile of the new tablet that reached the 100% of dissolved drug within 60 min vs. about the 40% obtained with the commercial tablet.

An improved drug bioavailability can be reasonably expected as a consequence of the increased drug solubility and dissolution rate. Moreover, the permeation enhancer properties of cyclodextrins, included methyl βCD derivatives, are well documented [33,34,35]. In particular, other authors observed an increased HCT permeability in the presence of βCD, by performing experiments on non-everted intestinal sac model [9]. Further in vivo studies have been planned in order to demonstrate the actual improvement of therapeutic efficacy of the new tablet formulation.

## 4. Conclusions

An exhaustive study of the interactions between HCT and several kinds of cyclodextrins, in combination with different preparation methods for binary systems resulted in the selection of the COE product with RAMEB as the most appropriate to improve drug dissolution properties, as a result of the best complexing-solubilizing ability of such CD and the complete drug amorphization achieved by co-evaporation in its presence. Solid-state studies of HCT in mixtures with three types of NCs at different w/w ratios led to the choice of SV at the 1:4 *w*/*w* ratio as the best for establishing effective interactions with the drug. Among the different techniques used for HCT:SV binary systems preparation, the co-evaporation showed the greater ability to improve drug amorphization and dissolution. 

Ternary systems prepared by co-evaporation with both selected carriers, evidenced a synergistic effect of CD and NC in enhancing drug dissolution properties, giving a two-fold and a 12-fold increase in drug solubility as compared with the binary HCT-RAMEB COE product and the pure drug, respectively, thus confirming the great potential of such a combined approach.

Tablets prepared with the selected ternary systems clearly showed a better dissolution profile as compared with a marketed formulation, with an approximate 60% increase of the drug amount dissolved at 60 min. Therefore, the new tablets give proof of their ability to strongly improve the HCT dissolution properties, thus, increasing the amount of drug available for oral absorption.

## Figures and Tables

**Table 1:** Stability constant (K_1:1_), complexation efficiency (CE), and solubilizing efficiency (SE) of complexes of the different examined cyclodextrins (CDs) with hydrochlorothiazide in pH 5.5 phosphate buffer at 25 °C.

CD Type	K_1:1_ M^−1^ ± s.d.	CE	SE ^a^
**βCD**	131 ± 2	0.27	2.3
**HPβCD**	106 ± 3	0.28	3.1
**HEβCD**	114 ± 1	0.23	3.3
**SBEβCD**	213 ± 4	0.44	4.7
**RAMEB**	234 ± 2	0.48	4.8

^a^ ratio between drug water solubility in presence of the highest CD concentration used (12.5 mM for βCD and 25 mM for other CDs) and alone.

**Table 2:** Thermal parameters and % residual drug crystallinity (% RDC) of hydrochlorothiazide (HCT) alone or in the presence of the different examined nanoclays (NCs).

NC Type	HCT:NC Ratio (*w*/*w*)	HCT Melting Peak (°C)	HCT ΔH_fus_(J/g)	% RDC
----	/	274.4	152.8	100.0
PHC	1:1	274.3	92.0	60.2
PHC	1:2	274.3	77.9	51.0
PHC	1:4	274.1	64.4	42.2
VHS	1:1	274.4	152.6	100.0
VHS	1:2	274.3	152.5	100.0
VHS	1:4	274.2	152.2	100.0
SV	1:1	274.4	84.2	55.1
SV	1:2	270.4	71.9	47.1
SV	1:4	269.2	56.5	37.0

**Table 3:** Thermal parameters and % residual drug crystallinity (% RDC) of hydrochlorothiazide (HCT) alone or in the different 1:4 *w*/*w* systems with sepiolite prepared by physical mixing (PM), solvent-sonication (SS), solvent magnetic stirring (SMS), co-grinding (GR), co-fusion (COF), solvent-heating (SH), and co-evaporation (COE).

Batch	Drug Melting Peak (°C)	H_fus_ (J/g)	% RDC
HCT	274.4	152.8	100.0
PM	269.2	56.5	37.0
SS	263.9	28.3	18.5
SMS	262.8	28.2	18.3
GR	260.4	15.4	10.1
COF	274.1	9.9	6.5
SH	274.0	8.2	5.4
COE	/	/	/

**Table 4:** Technological properties of the new tablet formulation and of the commercial reference tablet.

Tablet	Drug Content (%)	Diameter (cm)	Thickness (cm)	Weight (mg)	Hardness (N)	Friability (%)	Disintegration Time (min)
Esidrex^®^	98.5 ± 0.5	0.7 ± 0.0	0.25 ± 0.02	139.3 ± 1.9	4.0 ± 0.2	0.00 ± 0.00	6.0 ± 0.1
New tablet	99.2 ± 0.8	1.3 ± 0.0	0.20 ± 0.01	384.6 ± 0.5	6.6 ± 0.3	0.63 ± 0.01	13.0 ± 0.5

## Supplementary Materials

The following are available online at https://www.mdpi.com/1999-4923/12/2/104/s1, Figure S1: XRPD patterns of COE products with SBEβCD (yellow line), HPβCD (grey line) and HEβCD (orange line); Figure S2: Compatibility studies for tablet formulation.
