# Supplementary material for: Tablets of “Hydrochlorothiazide in Cyclodextrin in Nanoclay”: A New Nanohybrid System with Enhanced Dissolution Properties
Source: Pharmaceutics. 2020 Jan 28;12(2):104. doi: 10.3390/pharmaceutics12020104 (PMC7076548; doi:10.3390/pharmaceutics12020104)
Supplement: Supplementary file 1 [file pharmaceutics-12-00104-s001.pdf]

pharmaceutics

Supplementary Material: Tablets of "Hydrochlorothiazide in Cyclodextrin in Nanoclay": A New Nanohybrid System with Enhanced Dissolution Properties

Francesca Maestrelli, Marzia Cirri, Fátima García-Villén, Ana Borrego-Sánchez, César Viseras Iborra and Paola Mura

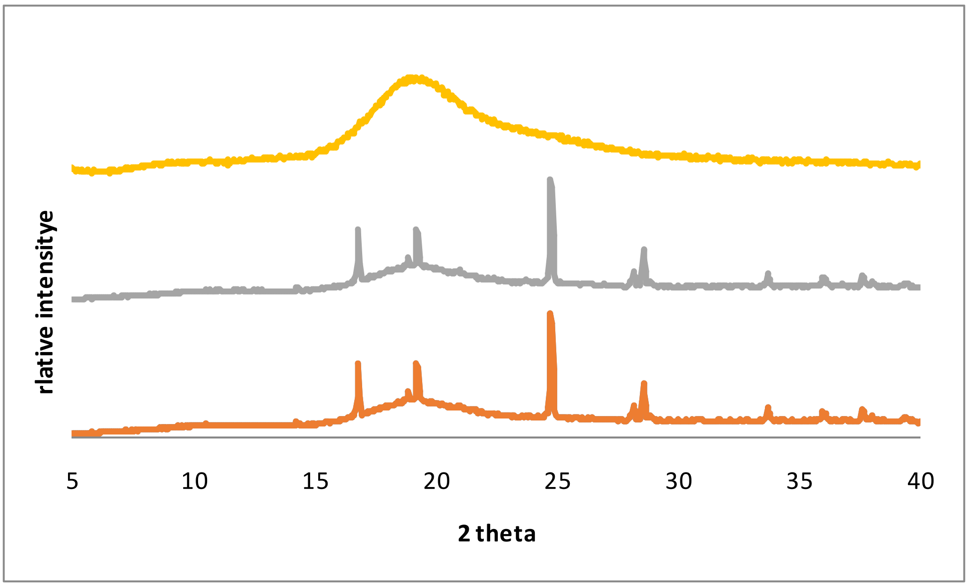

Figure S1. XRPD patterns of COE products with SBEβCD (yellow line), HPβCD (grey line) and HEβCD (orange line).

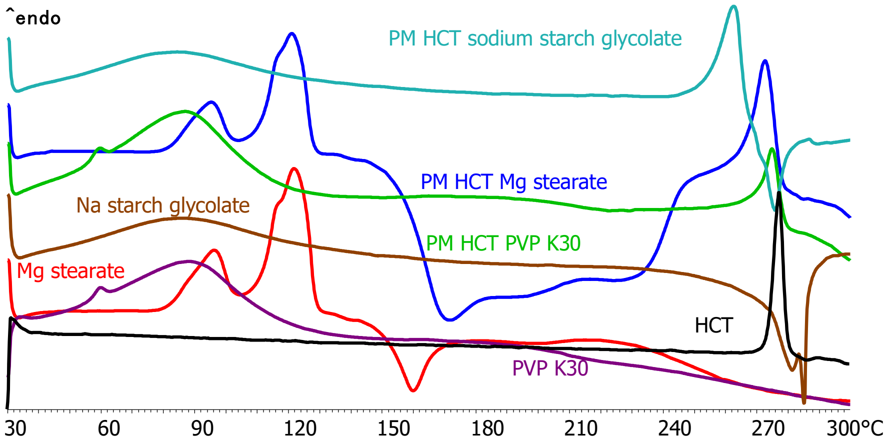

Figure S2. Compatibility studies for tablet formulation.
